# Using neurophysiological signals that reflect cognitive or affective state: six recommendations to avoid common pitfalls

**DOI:** 10.3389/fnins.2015.00136

**Published:** 2015-04-30

**Authors:** Anne-Marie Brouwer, Thorsten O. Zander, Jan B. F. van Erp, Johannes E. Korteling, Adelbert W. Bronkhorst

**Affiliations:** ^1^Perceptual and Cognitive Systems, Netherlands Organisation for Applied Scientific Research TNOSoesterberg, Netherlands; ^2^Team PhyPA, Biological Psychology and Neuroergonomics, Technical UniversityBerlin, Germany; ^3^Human Media Interaction, Twente UniversityEnschede, Netherlands; ^4^Training Performance Innovations, Netherlands Organisation for Applied Scientific Research TNOSoesterberg, Netherlands; ^5^Cognitive Psychology, VU UniversityAmsterdam, Netherlands

**Keywords:** passive BCI, physiological computing, mental state estimation, affective computing, neuroergonomics, EEG, applied neuroscience

## Abstract

Estimating cognitive or affective state from neurophysiological signals and designing applications that make use of this information requires expertise in many disciplines such as neurophysiology, machine learning, experimental psychology, and human factors. This makes it difficult to perform research that is strong in all its aspects as well as to judge a study or application on its merits. On the occasion of the special topic “Using neurophysiological signals that reflect cognitive or affective state” we here summarize often occurring pitfalls and recommendations on how to avoid them, both for authors (researchers) and readers. They relate to defining the state of interest, the neurophysiological processes that are expected to be involved in the state of interest, confounding factors, inadvertently “cheating” with classification analyses, insight on what underlies successful state estimation, and finally, the added value of neurophysiological measures in the context of an application. We hope that this paper will support the community in producing high quality studies and well-validated, useful applications.

## Introduction

A Brain–Computer Interface (BCI) has commonly been defined as a communication system in which messages or commands that an individual sends are encoded from brain activity as for example recorded through EEG (Wolpaw et al., 2002). In this view, a BCI is seen as an alternative communication channel that can supplement or replace common channels such as speech, typing, or gestures. While using such a BCI requires active involvement of the individual, brain signals can also be recorded without the need of conscious or effortful communication (the user remains passive in that respect). This is referred to as passive BCI (Zander et al., 2008; Zander and Kothe, 2011). A passive BCI represents an output channel above and beyond the more usual ones that can be used, possibly in combination with other physiological signals, to provide continuous information in real time about an individual's cognitive or affective state (or “mental state”). The question of how to detect and use information about mental state is also being approached from other fields of research. Fields that are related to passive BCI are physiological computing (Fairclough, 2009; Fairclough and Gilleade, 2014), affective computing (Picard, 1997), augmented cognition (Schmorrow et al., 2009), and neuroergonomics (Parasuraman and Rizzo, 2007). The focus of this paper is on the general area of research as investigated in all of these interrelated fields.

Several mental states have been shown, or suggested, to be reflected in neurophysiological signals, and several types of use have been proposed. Examples include monitoring cognitive workload through cardiovascular measures for adaptive automation (Stuiver and Mulder, 2014) or using Event-Related Potentials (ERPs) in response to errors to correct for an erroneous action (Chavarriaga et al., 2014). While passive BCIs make use of online neurophysiological responses, neurophysiological responses may also be used in an offline fashion. Examples of this include using measures of workload to evaluate different systems (e.g., interface designs) or using measures of stress to evaluate interventions taken to reduce mental stress (Brouwer et al., 2013b).

Our impression is that generally, there is a strong belief that mental states can be well-inferred from neurophysiological signals, and easily harnessed in applications. This belief seems to be partly based on conclusions that are not always warranted or on potentially problematic generalizations. While similar problems play a role in other research fields, there are two specific aspects of research on neurophysiological signals for mental state estimation that aggravate these problems.

Firstly, the field is highly interdisciplinary by nature which makes it very difficult to design, conduct, and evaluate studies correctly with respect to all their elements. To realize a successful, validated demonstrator of a system using mental state as estimated from neurophysiological signals, we need expertise in sensor technology (targeted at easily wearable systems), signal processing, mathematical modeling, experimental design, psychophysiology, systems design, engineering, and knowledge of the targeted user group or field. It is an enormous challenge for scientists to oversee the state of the art in all these areas of expertise, and to integrate it successfully in their research, or accurately judge the quality of research performed by others.

Secondly, as has been observed in related fields, non-experts unjustifiably tend to regard neurophysiological data as conveying an objective truth. Especially recordings from the brain possess an air of truth or objectivity that is often unfounded (Canli and Amin, 2002; Farah, 2002), as for instance in the suggestion that brain activity patterns reveal true emotional involvement, even if subjective reports indicate otherwise. Weisberg et al. (2008) asked a group of neuroscience experts and a group of non-experts to judge explanations of psychological phenomena. Explanations could be good or bad, and could contain no or irrelevant neuroscience information. They found that non-experts judged explanations with logically irrelevant neuroscientific information as more satisfying than explanations without. In particular bad explanations profited of the addition of irrelevant neuroscientific information indicating that for non-experts, neuroscience information could mask problems in explanations of psychological phenomena. With respect to neuroscience and education, Howard-Jones (2009) notes that brain-based educational ideas can be very popular in spite of the fact that their claims are not backed by scientific evidence. He argues for scientists to not only communicate skeptically amongst themselves, but also with the educational community. Researchers in the general field of applied neurophysiology should take this to heart and take care that they make a clear distinction between what can be concluded from their results now and what may eventually be possible. This is not only important when addressing the layman audience but also peers who may not be experts on all of the underlying expertise areas in the field.

**Figure 1:**
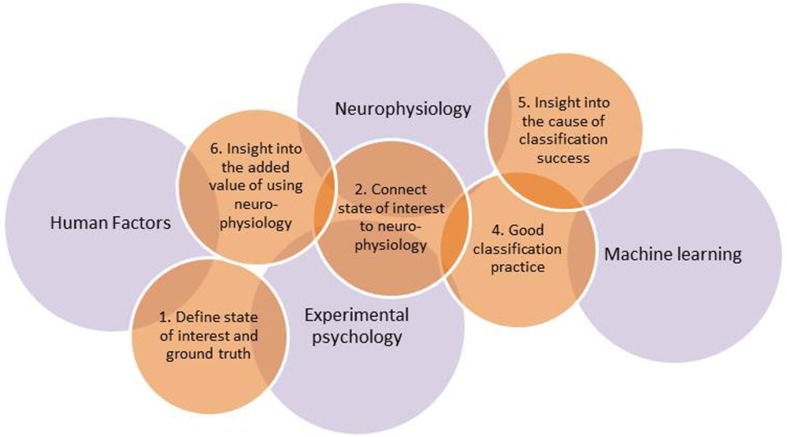
**Overview of five out of the six recommendations in relation to the major underlying fields**. Recommendation 3 concerning confounds is interweaved with all of the fields and all of the other recommendations (see Links between Recommendations).

Quantification of mental state is, thus, a popular but difficult art, requiring integration of knowledge across different scientific fields. Major overarching disciplines are neurophysiology, which provides knowledge on the functioning of the nervous system and how this can be measured, and experimental psychology, which provides methods to discriminate and assess mental states. Furthermore, one needs advanced classification algorithms developed within the field of machine learning, and, last but not least, human factors expertise to devise, develop, and test real-world applications. From our own stumbling over problems not in our direct areas of expertise, and from discussions with laymen as well as peers, we gathered that it would be useful to highlight a number of pitfalls that not only occur relatively often but that also may lead to unfounded conclusions and claims. An overview of earlier research, including our own work, resulted in six such pitfalls as well as six recommendations on how to avoid them. They are discussed below and may serve to improve the design and execution of a study as well as a checklist to keep in mind when reading and evaluating studies. A summary is provided in Table 1. An interesting, but not wholly surprising finding is that most of the pitfalls occur in interdisciplinary regions linking the four scientific fields mentioned above. This is illustrated in Figure 1 that represents 5 out of 6 recommendations associated with the pitfalls, and how they are linked with the underlying fields.

**Table 1:** **Summary of the six recommendations**.

**Recommendation**	**Key points**
1. Define your state of interest and ground truth	- Clarify how the state of interest and ground truth are operationalized
	- Examine multiple measures for determining ground truth (subjective, behavioral, knowledge of task or situation)
2. Connect your state of interest to neurophysiology	- Formulate hypotheses as to which neurophysiological measures are expected to vary in what way with the mental state of interest
3. Eliminate confounding factors (or at least, do not ignore them)	- Eliminate confounds by design
	- Examine *post-hoc* whether confounding factors occurred
	- *Post-hoc* selection of data to avoid confounds
	- Check whether neurophysiological data are more consistent with varying state (as hypothesized) or with effects of confounds
4. Adhere to good classification practice	- Take care that training data and test data are independent over time
	- Take care that choices in preprocessing and classification procedures are independent of validation data
	- Use proper statistical analyses to evaluate classification performance
5. Provide insight into the cause of classification success	- Present information about the way that neurophysiological processes underlying the different categories differ besides the classification results
	- Examine classification success of different (combinations) of features
6. Provide insight into the added value of using neurophysiology	- Explain that, and how, neurophysiological measures for mental state estimation potentially add value over using other (easier, cheaper) measures alone
	- Focus on applications that likely benefit from neurophysiological measures for mental state estimation

*Each recommendation and associated key points is more elaborately described in the corresponding subsections of Section Recommendations*.

We hope that our list of recommendations will be useful for researchers working on mental state estimation based on neurophysiological signals, especially for those entering the field, and will help to maintain high standards in both fundamental and applied studies. This paper also highlights the papers published in the current Frontiers Research Topic “Using neurophysiological signals that reflect cognitive or affective state.”

## Recommendations

### Define your state of interest and ground truth

To prevent confusion it is important to be clear from the outset about the mental state that you address. Mental states such as stress and workload usually have a long history in the (psychological) literature. Not all authors refer to the same concept when using the same word, which is partly dependent on the disciplines that researchers are coming from. It should be discussed how the addressed state has been defined in previous studies and which definition is adhered to in the study at hand. This may be guided by the specific application that the authors have in mind. It could be necessary to narrow down a specific concept (e.g., is the type of stress under investigation acute or chronic—Dickerson and Kemeny, 2004) or to refer to states that may underlie an overarching concept as is for example done for “engagement” in Fairclough et al. (2013). It is important to connect the mental state of interest to its operationalization in the study at hand, since this reflects what is being used or considered as the ground truth.

Obtaining ground truth can be extremely complicated in studies on mental states. Cognitive and affective states are not always easily detectable from behavior. This difficulty to observe them is precisely the reason why neurophysiological variables are of interest and of potentially added value. However, it also poses a problem when one wants to relate these “invisible” states to neurophysiology. One straightforward solution is to ask individuals to subjectively report their state, but this approach seems to conflict with the notion that neurophysiological correlates of mental state are valuable because of the fact that they are insensitive to (un)intended subjective distortion. This notion is reflected in the description of neurophysiological correlates of mental state as “objective measures.” However, it is not necessarily true that whenever a mismatch occurs between mental state as experienced subjectively and as indicated by neurophysiological measures, the latter is more reliable. Things are particularly fuzzy when it comes to mental states that are not experienced very clearly by individuals themselves. Even if you did not feel stressed, when your neurophysiological stress monitor says you are, it must be so (and you might even start to realize that actually you do feel kind of stressed)! Indeed, subjective reports cannot always be taken at face value, especially when individuals can be expected to be under pressure to provide a certain kind of answers (e.g., social desirability) or when subjective reports refer to experiences sometime in the past (Nisbett and Wilson, 1977). On the other hand, one could argue that, since affective states are effectively about the feelings of an individual, they are closer to the ground truth than neurophysiological correlates. For applications that claim to estimate mental state, the fuzziness of ground truth make it difficult to judge their validity on face value. Discrepancies between estimated mental state and subjectively experienced mental state could be due to errors of the application or due to the unreliability of introspection.

While determining ground truth of mental state is a difficult and perhaps unsolvable problem, validation of neurophysiological estimates can be performed to some extent by relating them to several different measures of mental state (Mauss and Robinson, 2009). Three broad categories can be distinguished. Firstly, behavioral measures such as button press accuracy (Mühl et al., 2014). Secondly, subjective measures such as responses on known scales for arousal and workload (Mühl et al., 2014) or for different types of emotion (Kashihara, 2014). Thirdly, knowledge of the condition that individuals are currently in, for example whether participants are performing a difficult or easy task (Mühl et al., 2014), whether a certain face stimulus has been paired with an aversive stimulus before or not (Kashihara, 2014), or whether individuals will undergo eye laser surgery in a few minutes or not in a study about stress (Hogervorst et al., 2013). Expert judgments can also be taken as, or contribute to, ground truth, where expert judgments can relate to all or some of the three categories. Expert judgments are also used in relation to neurophysiological measures (e.g., visual interpretation of neurophysiological measures is used to assess sleep stage: Huang et al., 2014).

### Connect your state of interest to involved neurophysiology

When trying to estimate cognitive or affective state based on neurophysiological signals, one aims to connect a certain psychological state to certain physiological signals. However, it is not a priori clear how, or to what extent, states defined from a psychological point of view map onto neurophysiology. Cacioppo and Tassinary (1990) describe four possible types of relation between psychological elements and physiological responses: one-to-one (i.e., one psychological element such as “attention” only maps to one physiological response, such as heart rate deceleration), one-to-many (attention also maps to skin conductance response), many-to-one (relaxation and attention map to heart rate deceleration) and many-to-many (attention and relaxation map to heart rate deceleration and skin conductance response). In her review on autonomic nervous system activity in emotion, Kreibig (2010) describes different views on how and to what extent physiological signals map onto emotions. While the experience of emotion goes together with physiological processes, one of the lessons to be taken from this review is that it is not only the emotion but also, or perhaps even mostly, the associated (future) action that defines the physiological response. Physiological processes have not evolved in order to provide signals that inform us about mental state, but in order to adapt and prepare the body for certain activity. This may be at the root of a counterintuitive finding that while (high arousal) fear will generally result in an increase of heart rate, presenting negatively valenced perceptual stimuli usually results in a decreasing heart rate (Bradley and Lang, 2000, see also discussion and results Brouwer et al., 2013c). This has been explained by the fact that while fearful pictures do not form a physical threat which would be followed by fight or flight, they do elicit heightened attentional processing which is associated with (a static posture and) a decreased heart rate (Lacey and Lacey, 1970; Graham, 1992; Codispoti et al., 2001). In a similar vein, sadness can be associated with autonomous signals associated with overt activity or withdrawn behavior resulting in a need to distinguish between “crying and non-crying sadness” (Kreibig, 2010). Reconceptualization of psychological and physiological processes may be required to design better models of the relation between mental states (psychological elements) and physiology (Cacioppo and Tassinary, 1990). Findings as described in the literature can be used to formulate hypotheses as to which neurophysiological measures are expected to vary in what way with the mental state of interest. This will help identify useful variables or features for training a mental state estimation classification model (see Section Adhere to Good Classification Practice). In addition, this knowledge can and should be used to examine whether the mental state estimation model is functioning as expected (see Section Provide Insight into the Cause of Classification Success).

### Eliminate confounding factors (or at least, do not ignore them)

When designing a study or evaluating an application, one should check whether the examined mental states of interest co-vary with other factors that a priori are not informative of the state of interest. If these confounding factors affect neurophysiological variables on their own, their effect can easily be mistaken by an effect of the state of interest.

A factor that can often potentially explain observed differences between mental states are differences in body movement. These body movements can, for instance, consist of button presses, turning a steering wheel, eye movements, or movements related to speech. Body movements can affect neurophysiological recordings through artifacts caused by subtle movements of the sensors or wires (Strait and Scheutz, 2014), by “real” effects on the neurophysiological signals such as electrical potential differences caused by rotating the eyes being reflected in EEG signals, or by “real” effects on the neurophysiology itself such as an increased heart rate or increased activity of the motor cortex when planning and executing movements.

Another type of confound is related to other mental states or processes co-varying with the state of interest. For example, when one aims to increase workload by increasing the number of visual stimuli, observed differences in EEG may not (only) be caused by a difference in workload but by the fact that the brain is processing more visual information in the one case than in the other (Brookings et al., 1996). Another often occurring example in this category is that studies aiming to find neurophysiological markers of emotions do not keep the arousal levels of the examined emotions equal, so that reported neurophysiological correlates of types of emotions may actually reflect levels of arousal (Oliveira et al., 2011).

Finally, it is important to realize that factors varying with time can have a huge impact on neurophysiological variables (e.g., Brouwer et al., 2014; Touryan et al., 2014). This can be a problem when experimental conditions co-vary with time relative to the onset of the study. If a difficult condition is presented before an easy condition, the higher heart rate in the former may not be related with task difficulty but with not yet being used to the experimental setting, previous physical activity or other unknown time related effects. Providing participants with practice or habituation time before the actual recordings start may alleviate time related effects.

From the perspective of fundamental science, the best way to deal with the effects of confounds is by the design of the study. Different conditions ideally only differ with respect to the examined state of interest. Different levels of mental state ideally should be present repeatedly, at different moments in time over the course of the recording session. However, complete control is not always possible or desirable. In ambulatory psychophysiological studies the aim is to investigate neurophysiological signals in the context of potential application or in contexts that are more ecologically valid than studies in the laboratory (Turpin, 1990; Picard, 1997). This usually conflicts with the attempt to control for confounds. When confounds cannot be avoided, examining the data can clarify whether or not found effects of mental state on neurophysiological variables are likely due to the varying mental state of interest or likely due to confounds. For instance, if the amount and type of body movements (speech, eye-, and hand movements) as indicated by suitable systems and measures are the same between conditions, they are an unlikely explanation for differences between conditions in neurophysiology (Betella et al., 2014). If there are differences, in some cases one could *post-hoc* select samples of data to fulfill this condition. For instance, when examining the differences between ERPs as elicited by looking at a target object vs. a non-target object in a search task where people were free to move their eyes, Kamienkowski et al. (2012) selected eye fixations in such a way that the preceding eye movement was the same in length and direction for both targets and non-targets. Hereby they ensured that differences between target- and non-target ERPs could not be explained by effects of differences in eye movements. Perhaps most importantly, and as touched upon before, neurophysiological differences between the examined states should be examined to check whether they fit the previously defined expected effects, or whether they hint at the effect of confounds. For example, when the goal is to distinguish between processing of stimuli in different auditory and visual modalities, data should at least show differential brain activity over the visual and auditory cortex (Putze et al., 2014). As another example, while one would expect differences in workload to be reflected by power changes in the alpha and theta band (Klimesch, 1999; Fink et al., 2005; Brouwer et al., 2012), differences in workload reflected by power changes in high EEG frequencies at frontal electrodes are likely caused by a difference in muscle activation (Whitham et al., 2007). This could mean that classification can be based on differences in frowning between low and high workload, rather than on differences in brain signals. If desirable, it is possible to correct to some extent for the likely contribution of muscle activation to classification results by excluding EEG high frequency features from the classification analysis (Dijksterhuis et al., 2013).

### Adhere to good classification practice

Classification analysis is an indispensable tool for estimating mental state, especially when high dimensional signals such as EEG signals are concerned. Traditional applied neurophysiology research typically uses group analyses to study neurophysiological correlates of mental state: neurophysiological variables and signals are averaged over multiple time intervals and individuals, and using statistical tools such as *t*-tests or ANOVAs it is determined whether varying mental state significantly affects neurophysiology. While this research and these methods are suitable to study the relation between mental state and neurophysiology in general, they would not suffice for a range of applied settings. If EEG frontal alpha power is significantly lower in high compared to low workload conditions for a group of experimental participants, we do not know whether this effect would be strong and consistent enough for estimating workload over a short time interval for a single individual. However, this is exactly what would be required if the information is to be used in adaptive automation. BCIs, that require short samples of brain signals of single individuals to be reliably translated into an intended action of a computer, welcomed classification techniques into the realm of neuroscience. These techniques had been successfully applied already in fields such as image and speech recognition. Lotte et al. (2007), van Gerven et al. (2009), Domingos (2012), and Lemm et al. (2011) provide easily readable reviews on classification. In short, (supervised) classification models are trained using samples of neurophysiological data that are labeled according to the states of interest (e.g., “low workload” and “high workload”). Subsequently, these trained models are used to label new, unseen neurophysiological data. If the label of this unseen data is known, the label as estimated by the classification algorithm can be compared to the actual label, and performance of the classifier can be determined. Subsequently, proper statistical analyses should be performed to interpret and evaluate classification performance (see e.g., Mueller-Putz et al., 2008).

While the classification procedure as described may seem simple enough, there is a multitude of options to be chosen and potential mistakes to be made that may lead to failure of successful classification or to overly optimistic results. Lemm et al. (2011) give a helpful summary of how machine learning is, or can be, unintentionally abused in brain imaging.

An important potential reason for overly optimistic results is that data used to train the model is not independent of data used to test the trained model. This links to the previously discussed effect of time-related factors (often referred to as non-stationarities). As an example, consider an experiment in which the level of workload is alternated in four blocks of 5 min. If a model is trained with 50% of 1-s randomly drawn samples, and tested using the remaining 50%, the classification success will be inflated since training and testing data from the same class are often close in time. Simply because of this fact, they will be similar rather than being similar because they originate from the same workload condition.

The number of free parameters that needs to be set when performing classification analysis is very large, varying from the type of classification algorithm and feature selection procedure, to criteria for outlier rejection and settings of hyper parameters specific to the exact pre-processing and classification procedures. This links to another important cause of inflated classification accuracy, which is reporting on pre-processing and parameter settings with the accompanying classification performance that happened to correspond to the best results. This good performance will partly be due to chance and not be reproducible. Whether or not this occurred for a specific study can be difficult to judge from information that is usually reported in a paper. It is important to choose such parameters separately from the test set that is used in the end to estimate classification performance (nested cross-validation—Lemm et al., 2011).

The problems as indicated above do not exist for studies that apply trained classification models in real time as now increasingly becomes the standard in BCI research, since in that case, making use of time related effects is not possible and classification performance is unmistakably associated with previously determined parameter settings.

### Provide insight into the cause of classification success

While classification analyses are very useful, they are essentially black box analyses. Data are used to train a model which subsequently turns out to be successful or not in properly classifying new data, but if it works it is largely unknown what is at the basis of success. It is therefore important to not only present information about classification results but also about the way that neurophysiological processes underlying the different categories differ. This provides a check as to whether the data are as expected or whether a confound could be responsible for classification success.

Note that results of traditional and classification analyses do not need to overlap exactly. For example, when a certain neurophysiological variable varies both with the mental state of interest and with time, a paired *t*-test can indicate a strong effect of mental state while most classification models based on the same information are expected to perform badly because of the time effects. This is because a traditional statistical procedure averages out general time effects while this is not straightforward for classification analyses. This difference between the two types of analyses is a possible explanation for the counterintuitive finding in an experiment that varied workload using a task that changed difficulty level every 2 min, for 24 times. While heart rate was a poor feature in workload classification analysis (Hogervorst et al., 2014), an ANOVA indicated that heart rate very reliably increased with mental workload (Brouwer et al., 2014). For these data, a strong decrease in heart rate over the time course of the experiment was found (Brouwer et al., 2014). This effectively means that a sample of high heart rate data could originate from the start of the experiment or from a high workload condition.

Reversely, classification analysis could indicate that certain features are very informative for estimating mental state, while this does not seem to be the case when examining results of traditional analyses. This can be the case when the way that certain neurophysiological features are associated with mental state is very different for one individual compared to another, while the association is very consistent within individuals. This has been suggested to be the case for EEG signals associated with workload (Grimes et al., 2008). It may also hold when examining a range of different neurophysiological responses to complex situations that allow for different strategies or coping styles. The announcement of a camera crew arriving in 2 min to take an interview about your research may lead to some kind of stress for virtually all people, but while one individual will quickly and intensely start thinking about the messages she wants to convey, the same event will elicit a pure fright response in the other.

Combining the two types of analyses gives us insight in the neurophysiological processes underlying cognitive and affective states. A strong association of a neurophysiological variable with mental state according to a traditional approach indicates that there is a reliable association in the same direction across individuals (possibly on top of other factors that play a role but that are equally strongly present in the different mental states, e.g., time varying factors). A strong association of a neurophysiological variable with mental state according to an individually tailored classification analysis indicates that there is a strong association in the same direction within that individual, and that the variable is not strongly affected by other factors or occurring events. Offline classification analyses based on different (combinations of) features are arguably the most straightforward approach for finding out exactly which features contribute most (Hogervorst et al., 2014). Other methods like independent component analysis (ICA; Makeig et al., 1996) or transforming the classification backward model into a forward model (Haufe et al., 2014) can lead to a spatial interpretation of the signal.

### Provide insight into the added value of using neurophysiology

Carefully controlled experiments allow us to verify which neurophysiological measures are connected to mental state and in what way. However, in an applied research field where we want to use these signals, this is not enough. It should also be pointed out under which circumstances and in what way these signals are envisioned to be helpful. This is not always easy since one should realize that usually, there are alternative ways to retrieve the desired mental state information. Why would one use neurophysiological signals to estimate how well people like a product if they can also be asked—is there empirical evidence that this will help to better predict which product they will buy? Why would one use neurophysiological signals to estimate driver's workload if distance to the lines on the road is informative as well? While the idea of passive BCI is that we have a channel of information available “for free” since users do not need to spend attention or conscious effort to convey information about their mental state, there are costs involved with respect to buying and wearing the sensor equipment, calibration procedures, etc. Relative to other sources of information, these costs can (as of yet) be quite high, especially for brain signals. It should thus be explained that, and how, they potentially add value. Note that this could also be in the context of a combination of different types of information. See for example Huang et al. (2007) who combined ERP and button press responses for detecting targets within series of rapidly presented images, and Lin et al. (2014) who combined characteristics of music with listeners' EEG to estimate emotion.

#### Confounds

In real life applications, the discussion on confounds touches the discussion on alternative sources of information about cognitive and affective state. The confounds that we carefully want to exclude in experimental situations in order to verify that and investigate how neurophysiology is connected to mental state, are abundant in real life and can actually be used if these confounds are reliably present in both the model training and the application data. For instance, if a high workload situation in air traffic control reliably co-varies with more arm movements because of button presses, more detailed information presented on the screen, and more verbal communication compared to a low workload situation, EEG is expected to co-vary with workload because of movement artifacts as well as activity in the visual cortex, and breathing variables are expected to co-vary with workload too. Thus, EEG and breathing can be used to estimate the workload situation that the controller is in. This is so because even though in principle, movement artifacts, visual information processing and speech are unrelated to workload, they *are* related for the specific situation at hand (air traffic control). In such cases, one should take care to define conclusions or claims properly (i.e., such as not to claim that differences in EEG are caused by different brain processes associated with workload where actually, it is a difference in movement that is responsible for the effect). Secondly, it should be realized that the trained model will not generalize to workload situations with other sensory input and motor output than the examined situation. Finally, an important question to answer when confounds are responsible for the effect is whether it is conceivable that neurophysiological data will improve mental state estimation over and above estimation based on measuring the confounding information in an easier way. For instance, the number of button presses, the information presented on the screen and a microphone indicating whether the air traffic controller is talking could in this case be just as or more effective to estimate workload than EEG and easier to collect.

#### Characteristics of applications that likely benefit from neurophysiological measures for mental state estimation

In general, applications using information about mental state as estimated on the basis of neurophysiology are likely to be of added value if firstly, alternative measures of mental state are not available, unreliable or difficult to obtain, and secondly, mental state as estimated by neurophysiological signals is relatively reliable. Relatively reliable information from neurophysiological signals is expected when there is little noise due to body movements and when estimates can be based on large amounts of data. Also, relatively reliable information is expected when the relation between neurophysiology and the mental state of interest is clear and well-established. Fundamental neurophysiological studies, even if only based on group effects, indicate which neurophysiological variables reflect which mental state. Emotional valence and arousal can be indicated by peripheral measures such as heart rate and skin conductance (Mauss and Robinson, 2009; Brouwer and Hogervorst, 2014; van der Vijgh et al., 2014). Workload has been shown to be measurable through EEG (even though for many studies, effects may be completely or partly caused by confounds—see Gerjets et al., 2014 for a discussion on this). Fatigue and behavioral lapses have been shown to relate to EEG signals (Wang et al., 2014) and variables related to eye blinks (Schleicher et al., 2008). Errors can be detected by analyzing event-related potentials occurring after an error on a single-trial basis (Chavarriaga et al., 2014). In addition, active BCI studies for which (consciously modifiable) signals with a high signal to noise ratio are of paramount interest, indicate which mental states can be measured at the level of a single person at one moment in time. For instance, motor imagery based active BCIs (Kalcher et al., 1996; Wolpaw et al., 2002) show that it is relatively easy to distinguish EEG signals that co-occur with imagining right hand movement (power decrease or desynchronization in the 8–13 Hz band over the left sensorimotor cortex) from those that co-occur with left hand movement imagination (power decrease in the 8–13 Hz band over the right sensorimotor cortex). Pineda et al. (2013) show that a similar principle can be used to distinguish sounds that are related to hand-based action from sounds that are related to mouth-based action. In general, brain activity in certain brain areas is roughly indexed by power in the 8–13 Hz frequency band, where high power indicates functional cortical inhibition (reviewed by Klimesch, 2012; Horschig et al., 2014). This inhibition has been proposed to block task-irrelevant processes, therewith enhancing task-relevant brain processes in other brain areas. When measured over the visual cortex, this even allows to estimate spatial direction of visual attention which may be used in active BCI (Bahramisharif et al., 2010). Another important class of BCIs is based on the P300 ERP (Farwell and Donchin, 1988). The P300 is also related to attention—it occurs after an event that is relevant for and attended by an individual. The distinction between attended and non-attended stimuli can be made on a single ERP basis. This is also true for ERPs starting at fixation onset rather than stimulus onset in the context of a visual search task, where distinguishing between targets and non-targets on the basis of fixation-locked ERPs tended to outperform distinguishing between targets and non-targets on the basis of fixation duration (Brouwer et al., 2013a).

#### Examples of existing and promising applications

A concrete example of an application that fulfills the criteria of no or unreliable alternative measures of the mental state of interest and reliable neurophysiological signals, is “concealed information detection” (Farwell et al., 2014). In this work ERPs are used to determine whether or not it is likely that a person under “criminal” investigation possesses certain information. This is done by examining whether ERP responses to this concealed information are more similar to responses to known and relevant information, or more similar to responses to irrelevant information. In this case, verbal information about not knowing the concealed information cannot be taken at face value, i.e., there is no reliable alternative information present. In addition, data can be collected such that little noise is present because of body movements. Large amounts of data can be collected and analyzed offline, and the procedure is based on the established differential effect of relevant and irrelevant stimuli on ERPs. Another promising example from the current special issue is studied by Wang et al. (2014). They showed that EEG indicates whether a warning, that individuals respond to behaviorally, actually alerted them or not as indicated by a quick vs. late behavioral response later on. In contrast to EEG, the behavioral response to the warning did not reveal an individual's alertness level, i.e., neurophysiology is likely to add value. Furthermore, fatigue or alertness has been well-demonstrated to be associated with neurophysiology, and Wang et al. propose an application in driving, where little body movement occurs. They also show that similar results were obtained in an experiment using lightweight, portable, and low density EEG equipment. Another application area that at least fulfills some of the requirements is evaluating working with different interfaces and displays with respect to workload and attention. For this, performance and subjective measures could be used as well, but neurophysiological measures could provide more continuous information, such that potential difficulties could be pinpointed more exactly. Analysis can be performed offline. In certain real-life evaluation scenarios, neurophysiology may add value because social demand characteristics could play a role, e.g., when individuals are reluctant to report that an ad within a display caught their attention. Real time prediction of errors based on neurophysiological correlates of workload or drowsiness could be helpful, especially if people are unable or reluctant to signal high drowsiness or workload by themselves and if other behavioral performance measures are not available (e.g., for monitoring images from a surveillance camera where relevant events seldom occur but a single miss could have serious consequences). A potential application that builds upon well-established motor imagery BCI as discussed in the previous section, is detecting and feeding back information on motor imagery to support motor rehabilitation (Mokienko et al., 2014).

There are applications using or claiming to use neurophysiological signals for estimating mental state that are successful up to the point of commercial success, while they do not fulfill the proposed conditions as to the unavailability of reliable alternative measures of mental state or to the reliability of mental state as estimated by neurophysiological signals. In these applications, the (apparent) use of neurophysiological signals is perceived by the user as fun or helpful in itself. An example of this are the moveable “Necomimi cats ears” that users can wear on their head. It is claimed that the device reflects the emotions of the wearer based on EEG as measured by a single dry electrode on the forehead. Also, there is a range of biofeedback and neurofeedback games commercially available that are said to provide users with information about their neurophysiological signals so that they can learn to adapt these, which in turn should lead to improved health or well-being. While good research is conducted in the neuro- and biofeedback area (e.g., van Boxtel et al., 2012; see also Frontiers Research Topic “Learned brain self-regulation for emotional processing and attentional modulation: from theory to clinical applications” e.g., Enriquez-Geppert et al., 2014), double-blind controlled research is scarce (Vollebregt et al., 2014). For the commercially available self-help applications it is unclear that these benefit the user over a placebo effect. Even though work in the area of commercial consumer applications is not up to high scientific standards yet, it is valuable and important for advancing knowledge in wearable, low energy equipment, user acceptance, and usability. However, care must be taken not to mislead users on what the equipment exactly does and achieves.

### Links between recommendations

While we grouped and presented pitfalls and recommendations as six separate entities, they are closely interconnected. Most clear in this respect is the recommendation concerning confounds (3), that runs through all other recommendations. Confounds should be recognized when defining ground truth (see recommendation 1). One should consider that classification results may not reflect differences in mental state but rather result from confounding factors (4 and 5). Recommendation 2 on connecting the state of interest to involved neurophysiology could help determine whether an effect is likely based on a confound or not. Finally, one should consider to actually make use of confounds, i.e., regard and use them as sources of information (see recommendation 6). Another example of interconnections are that experimental skills as described under recommendations 1 and 3 are important to derive valid training data for the classification algorithm (4 and 5). Finally we would like to mention that connecting the state of interest to involved neurophysiology (2) is important to choose sensible features to train the classifier (4), where good data-driven classification practice may also lead to improved understanding of the mapping between mental state and neurophysiology (Lemm et al., 2011) (2).

## Concluding remarks

A large body of previous research shows that in principle, neurophysiological variables contain information about mental state. Continuous knowledge of mental state could potentially be helpful in a range of application areas such as gaming, security, health, and mobility (van Erp et al., 2012). Two areas of research can be defined that are crucial for future success of applications making use of mental state estimation based on neurophysiology. These are sensor technology and generalization of mental state estimation across time, tasks, and people.

Advances in wearable, even fashionable sensor technology boosted the field and are expected to contribute further even though there are still major challenges to overcome. Currently, there are a number of dry electrode EEG systems available or under development. These systems do not require the application of gel and sometimes come with a fancy headset. While at least some of these systems approach performance of conventional wet systems (Zander et al., 2011; Chi et al., 2012), dry electrodes need pressure to overcome the lack of gel which can be uncomfortable. Debener and colleagues follow a different route by focusing on tiny lightweight electrodes that provide signals that are resistant to body movements such as those caused by walking (De Vos et al., 2014). For physiological signals besides EEG, wearable sensor developments are quick. Breathing and heart rate can be monitored without attaching sensors but by using a camera (Wu et al., 2012; Brouwer and Hogervorst, 2014) or radar (Lazaro et al., 2010), and wristbands that record heart rate or skin conductance are commercially available. Validation of these new types of equipment by independent parties is required.

For practical applicability, it is important that estimates of mental state based on neurophysiological signals can be generalized across tasks, time and individuals. The recommendations as discussed (e.g., the recommendation in Section Provide Insight into the Cause of Classification Success) are partly connected to improving generalization and to be able to predict whether, and under which circumstances, generalization is possible. For some types of signals and tasks such as the P300 in a P300-BCI, generalization (in this case, across days or even months) has been demonstrated not to pose a large problem (McCane et al., 2014). Wang et al. (2014) discuss that for EEG signals associated with fatigue, or (upcoming) lapses in performance, findings are similar across tasks. However, for many passive BCI-like applications it is difficult to create a training situation that can be used to train a classification model and that is sufficiently similar to the application situation. A workload classifier trained using known labels of a working memory task that varies in difficulty may not be able to estimate workload in a driving task where properties of the task and environment are very different. The present issue features a number of studies that worked on generalization across tasks. Stikic et al. (2014) show similarities in (unsupervised) neural network results trained and tested on neurophysiological data from combat marksmanship and golf putting tasks. Gerjets et al. (2014) propose a strategy to deal with cross-task generalization (see also Walter et al., 2013). The present issue also includes work on generalization across specific electrode montages and days (Estepp and Christensen, 2015). Touryan et al. (2014) modeled time related changes in EEG. Algorithms that can adapt the classification model on the fly could prevent problems due to generalization across time (e.g., Millán, 2004; Kindermans et al., 2012). Casson (2014) shows that adding artificial noise to EEG data helps to make classification performance more robust across time. Reuderink (2011) discusses generalization issues with respect to variability within and between users, and potential ways to make classification algorithms more robust which may help to reduce other generalization problems as well.

We would like to end this paper by stressing the importance of real-life studies. While laboratory studies designed to reveal the exact connection between mental state and neurophysiology are important, in an essentially applied field of research, we also need to design applications and test whether they have added value. This should also be done under real life circumstances rather than (only) in a laboratory. Individuals are expected to function differently in controlled lab environments and under ecological, every-day circumstances. For instance, compared to having images imposed at static eyes, visual information processing seems to be quicker when actively sampling the environment through eye movements in which case the brain “knows” when information processing of a new image will start (Kamienkowski et al., 2012). However, because of confounds in real-life studies, it will be hard to connect neurophysiological results directly to cognitive and affective state. Therefore, special care is needed with statements about cause and effect. Also in real-life studies, it is possible and desirable to investigate the likely cause of classification success. This will improve our understanding of the connection between mental state and neurophysiology as well as providing clues for alternative (potentially easier measurable) informative variables. Ultimately, what needs to be shown is that applications based on mental state estimation through neurophysiological signals support users and improve performance or well-being over and above the use of a sensible comparison application. This will likely refer to a certain context and a defined range of function (Fairclough, 2009). The field of mental state estimation through neurophysiological signals as a science will benefit from careful behavior of scientists as to statements of what is possible and potentially helpful and what not, as well as from high quality studies that avoid the most common pitfalls.

### Conflict of interest statement

The authors declare that the research was conducted in the absence of any commercial or financial relationships that could be construed as a potential conflict of interest.
